# Pirfenidone alleviates cardiac fibrosis induced by pressure overload via inhibiting TGF‐β1/Smad3 signalling pathway

**DOI:** 10.1111/jcmm.17478

**Published:** 2022-07-21

**Authors:** Na Li, Weijian Hang, Hongyang Shu, Ning Zhou

**Affiliations:** ^1^ Division of Cardiology, Department of Internal Medicine, Tongji Hospital, Tongji Medical College Huazhong University of Science and Technology Wuhan China; ^2^ Hubei Key Laboratory of Genetics and Molecular Mechanisms of Cardiological Disorders Wuhan China

**Keywords:** cardiac fibrosis, cardiac remodelling, pirfenidone, pressure overload, transforming growth factor‐β1

## Abstract

Cardiac fibrosis critically injured the cardiac structure and function of the hypertensive patients. However, the anti‐fibrotic strategy is still far from satisfaction. This study aims to determine the effect and mechanism of Pirfenidone (PFD), an anti‐lung fibrosis medicine, in the treatment of cardiac fibrosis and heart failure induced by pressure overload. Male C57BL/6 mice were subjected to thoracic aorta constriction (TAC) or sham surgery with the vehicle, PFD (300 mg/kg/day) or Captopril (CAP, 20 mg/kg/day). After 8 weeks of surgery, mice were tested by echocardiography, and then sacrificed followed by morphological and molecular biological analysis. Compared to the sham mice, TAC mice showed a remarkable cardiac hypertrophy, interstitial and perivascular fibrosis and resultant heart failure, which were reversed by PFD and CAP significantly. The enhanced cardiac expression of TGF‐β1 and phosphorylation of Smad3 in TAC mice were both restrained by PFD. Cardiac fibroblasts isolated from adult C57BL/6 mice were treated by Angiotensin II, which led to significant increases in cellular proliferation and levels of α‐SMA, vimentin, TGF‐β1 and phosphorylated TGF‐β receptor and Smad3. These changes were markedly inhibited by pre‐treatment of PFD. Collectively, PFD attenuates myocardial fibrosis and dysfunction induced by pressure overload via inhibiting the activation of TGF‐β1/Smad3 signalling pathway.

## 1 INTRODUCTION

Hypertension is the most common cause of heart failure worldwide.^1^ Cardiac remodelling, including cardiac fibrosis and hypertrophy, is an essential pathological change during the development of heart failure.^2^ Heart failure with preserved ejection fraction (HFpEF) is largely characterized by aggravated cardiac fibrosis, which mainly damages myocardial compliance.

Cardiac fibrosis is characterized by excess proliferation of cardiac fibroblasts (CFs) and trans‐differentiation to myofibroblasts (MFs), lessening extensibility of myocardium and injuring cardiac function.^2^ Angiotensin converting enzyme inhibitors (ACEIs) were used for anti‐cardiac remodelling induced by pressure overload, including Captorpril (CAP),^3^, ^4^, ^5^ Perindopril,^6^ and so on. CAP was chosen as a positive control in this study due to its remarkable effect on inhibiting TAC‐induced heart failure.^7^ Pirfenidone (PFD), widely used for treating idiopathic pulmonary fibrosis, has been proven to attenuate renal, liver, and lung fibrosis.^8^, ^9^ PFD and CAP exert different mechanisms of anti‐myocardial fibrosis. CAP attenuates heart failure via inhibiting Wnt3a/beta‐catenin and Jak2/Stat3 pathways.^7^ However, PFD attenuated cardiac fibrosis induced by thoracic aorta constriction (TAC) via suppressing Nod‐like receptor pyrin domain containing‐3 (NLRP3) inflammasome formation.^10^ In addition, lung fibrosis was alleviated by PFD through inhibiting profibrotic signalling pathways, including transformation growth factor‐β1 (TGF‐β1)/Smad3 pathway and mitogen‐activated protein kinase (MAPK) pathway.^8^, ^11^ Smad‐dependent and Smad‐independent signalling activated by TGF‐β1 are both involved in cardiac fibrosis.^12^ However, whether PFD inhibiting the pressure overload‐induced cardiac fibrosis was linked to inhibiting TGF‐β1/Smad3 signalling has never been elucidated. Therefore, this study was to validate the effect and exploit the related mechanism of PFD in preventing cardiac fibrosis induced by pressure overload.

## 2 METHODS

### 2.1 Materials

Agents used in this study including PFD (300 mg/kg/day, Kangdini, Beijing), Captopril (CAP, 20 mg/kg/day, MedChemExpress), antibodies against α‐smooth muscle actin (α‐SMA, ABclonal, 1:1000), Vimentin (ABclonal, 1:1000), TGF‐β1 (ABclonal, 1:1000), phosphorylated‐TGFβ receptor 1‐S165 (p‐TGFβR1^S165^, Affinity Biosciences, 1:1000), TGFβ receptor 1 (TGFβR1, ABclonal, 1:1000), phosphorylated‐Smad3‐S423/425 (p‐Smad3^S423/S425^, ABclonal, 1:1000), total Smad3 (ABclonal, 1:1000), Smad7 (ABclonal, 1:1000), glyceraldehyde‐3‐phosphate dehydrogenase (GAPDH, ABclonal, 1:10,000) were all commercially purchased.

### 2.2 Animal study

C57BL/6 (8‐week‐old, male, SPF grade)^13^, ^14^ were commercially provided. All animal experiments were approved by the National Institutes of Health Guide for the Care and Use of Laboratory Animals and approved by the Institutional Animal Use and Care Committee at the Huazhong University of Science and Technology, China.

After 1 week of adaptive feeding, the mice were randomly divided into six groups: Sham group, PFD group, CAP group, TAC group, TAC + PFD group, TAC + CAP group. The mice were anaesthetised and subjected to sham or TAC surgery with a 27‐gauge needle and 7–0 silk suture as described previously.^15^ Mice in the sham group and the TAC group were provided by Vehicle, PFD (300 mg/kg/day)^16^ and CAP (20 mg/kg/day),^7^ respectively. Vehicle, PFD, and CAP were intragastric administrated with an equal volume (200 μl). 6–7 mice in each group were treated for 8 weeks.

### 2.3 Transthoracic echocardiography

The echocardiography was conducted after an 8‐week treatment to examine cardiac function as described before.^17^ The mice were anaesthetised with isoflurane gas and placed on an ultrasonic testing table at a constant temperature of 37°C. Their limbs were fixed on the limb lead electrode, and were continuously anaesthetised with a low dose of ether. The heart rate of mice was controlled at about 500–600. After removing the fur on the chest wall, the mice were routinely disinfected. Coupling agent was coated on the chest wall above the heart, and the morphological changes of the heart were detected at the positions of the long axis, short axis and four‐chamber heart with an ultrasonic probe. Morphological and functional indicators including the left ventricular posterior wall thickness in diastole and systole (LVPWd and LVPWs), left ventricular anterior wall thickness in diastole and systole (LVAWd and LVAWs), left ventricular internal dimension in diastole and systole (LVIDd and LVIDs), left ventricular mass (LV Mass), ejection fraction (EF), and fractional shortening (FS) were recorded.

### 2.4 Histological measurement

The hearts were harvested after 8‐week treatment and embedded with paraffin. The hearts were sectioned at 5‐μm thickness and subsequently stained with Haematoxylin–eosin (H&E) staining and Sirius Red (SR) staining for histological and fibrotic quantitative changes, respectively. The myocyte cross‐sectional area (CSA) and the perivascular and interstitial fibrosis were measured by a quantitative digital image analysis system (Image‐Pro Plus 6.0). The analysis was performed in a minimum of 6 hearts for each experiment group, and 5 visual fields were measured in each sample. The expression and distribution of α‐SMA and Vimentin were examined by immunohistochemistry (IHC).

### 2.5 Isolation and culture of primary cardiac fibroblasts

Six‐week‐old C57BL/6 adult mice were sacrificed to isolate CFs. Briefly, all mice were killed by cervical dislocation, the ventricles were harvested and cut into ~1 mm^3^ pieces in a dish with ice‐cold PBS. Subsequently, the ventricle tissues were digested using digestive enzyme, which was composed of 0.125% trypsin and 0.05% collagenase type II dissolved in sterile PBS, at 37°C five times for 7 min each to dissociate cardiomyocytes and CFs. The cells were centrifuged, resuspended and incubated for 2 h in a 100‐mm dish to allow CFs to attach readily to the bottom of the culture dish.

### 2.6 Cell viability assay

The cell viability was detected by Cell Counting Kit‐8 (CCK8, Med Chem Express) assay. Primary CFs were seeded in 96‐well growth‐medium plates overnight at 1 × 10^4^ cells/well. After 24 h, the medium was replaced, and cells were stimulated with different concentrations of PFD (2 × 10^−3^, 4 × 10^−3^, 8 × 10^−3^, 16 × 10^−3^). Next, the cells were maintained at 37°C and 5% CO_2_ in a humidified incubator for 24 h, then these cells were cultured with CCK‐8 for 1 h. Absorbance values were examined with 450 nm wavelength.

### 2.7 Immunofluorescent staining

As described previously,^18^ CFs were fixed directly with 4% paraformaldehyde at room temperature for 10 min. Goat serum (10% in 0.1% TritonX‐100 PBS) was used to block nonspecific binding. Then, intended primary antibodies were added to the section or cell at 4°C overnight. The fluorescent secondary antibodies were used to visualize the corresponding subsets. Cells were stained in 4,6‐diamidino‐2‐phenylindole (DAPI) for 5 min and observed under a fluorescence microscope (Carl Zeiss, Axio Imager.A2).

### 2.8 Western blot

Total ventricular and cytoplasmic proteins were extracted from the LV myocardium and cultured fibroblasts, respectively. Tissues and cells were homogenized in cold RIPA lysis buffer supplemented with Complete Protease Inhibitors cocktail (Roche). Protein concentrations were measured using a BCA protein assay kit (Beyotime Institute of Biotechnology). Protein samples were separated by sodium dodecyl sulfate‐polyacrylamide gel electrophoresis (SDS‐PAGE) and then transferred to polyvinylidene difluoride (PVDF) membranes (Beijing Solarbio Science & Technology Co, Ltd.) by electroblotting. After being blocked with 5% skim milk for 1 h, the membranes were incubated with primary antibodies (1:1000) overnight at 4°C, and then incubated with HRP‐conjugated Affinipure Goat Anti‐Rabbit IgG (H + L) (ABclonal) (1:10,000). After 1 h, bands were detected with Immobilon Western HRP Substrate (Abclonal), and the blots were analysed and quantified by densitometry using the ImageJ program.

### 2.9 Statistical analysis

All experimental data were analysed by the SPSS 19.0 statistical software (Armonk), and expressed as the mean value ± the standard error (x ± SEM). *T*‐test and one‐way analysis of variance (anova) performed using Prism 5 (GraphPad Software) were used for Two‐group and multiple group comparisons, respectively. *p* < 0.05 was considered statistically significant.

## 3 RESULTS

### 3.1 Pirfenidone attenuated cardiac dysfunction induced by TAC in mice

**FIGURE 1:**
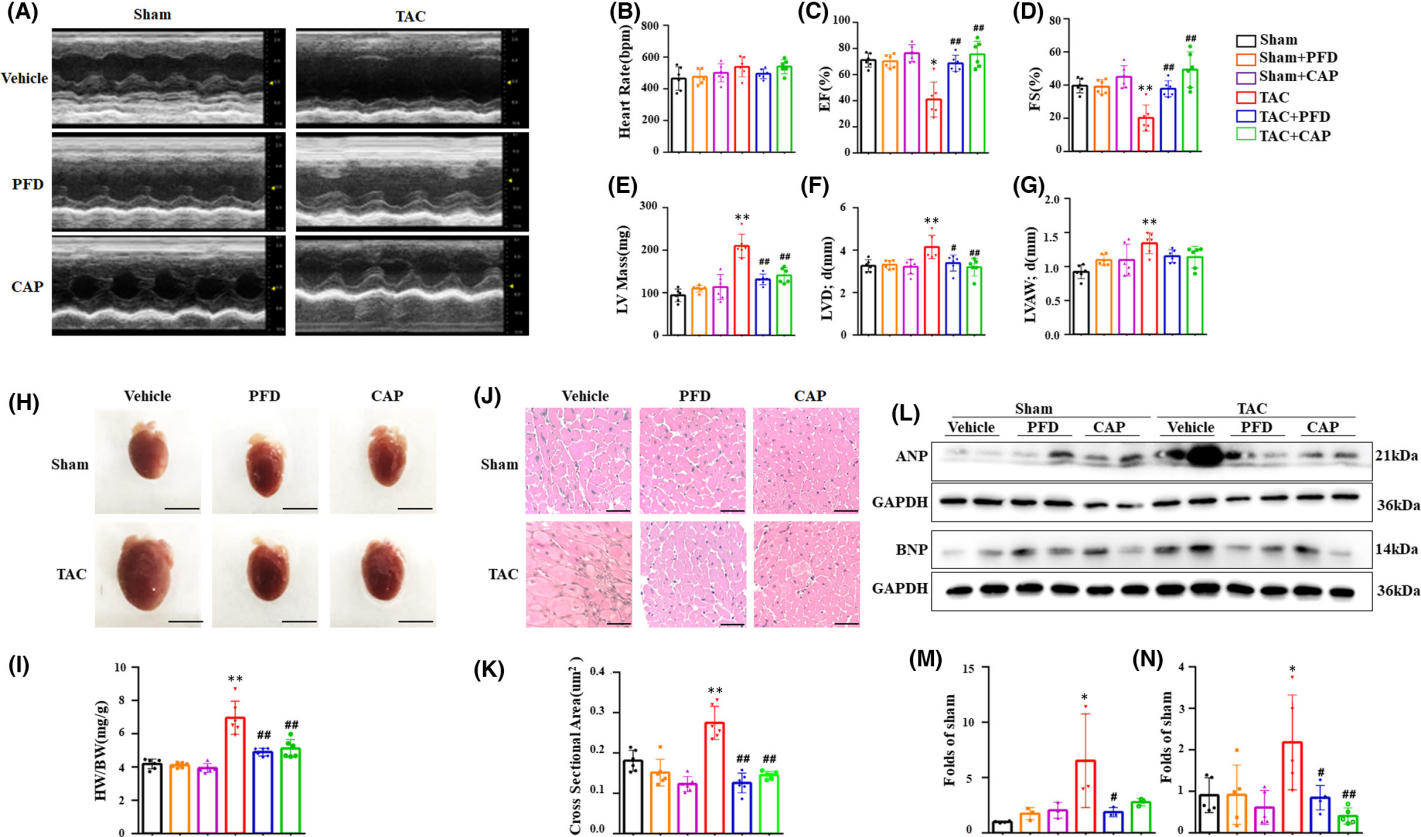
Pirfenidone attenuated cardiac dysfunction induced by TAC in mice. (A) Representative echocardiographic graphs. (B–G) Echocardiographic analysis 8 weeks after TAC (*n* = 6). (H, I) Gross morphology of mouse heart and HW/BW ratio. Scale bar = 5 mm. (J, K) Representative H&E staining images and cross‐sectional area (CSA) of cardiomyocytes. Scale bar = 5μm. (L–N) Western blotting and relative expression of ANP and BNP in the indicated mice. *p < 0.05, ** *p* < 0.01 vs. Sham group; ^#^ *p* < 0.05, ^##^ *p* < 0.01 vs. TAC group

Significant cardiac remodelling and dysfunction were caused by 8‐week‐TAC in C57BL/6 mice, which was proven by representative echocardiography images (Figure 1A) and parameters (Figure 1B–G). As shown, given the similar heart rate of mice, EF in the TAC group was strikingly decreased to 57.6% of the sham group, and FS was decreased to 50.1%. LV mass, LVIDd and LVAWd were increased in the TAC group to 2.2, 1.3 and 1.5 folds of the sham group, respectively. However, PFD and CAP reversed these deteriorations. Compared to the TAC group, EF was increased by 27.6% and 34.6%, FS was increased by 17.6% and 29.1% after PFD and CAP interference, respectively. Conversely, LV mass and LVIDd were significantly decreased after PFD and CAP treatment. Gross morphology and heart weight/body weight (HW/BW) ratio (Figure 1H,I) demonstrated that TAC caused eccentric cardiac hypertrophy, consistently with the enlarged heart chamber showed in Figure 1A. H&E staining (Figure 1J,K) was used to examine CSA of cardiomyocyte. The CSA of cardiomyocytes in the TAC group was increased by 1.5 folds relative to the sham group, while the CSA of mouse heart in the TAC + PFD and the TAC + CAP group were reversed to 45.7% and 52.8% of the TAC group, respectively. Furthermore, the expression of atrial natriuretic peptide (ANP) and brain natriuretic peptide (BNP) (Figure 1F–H), the markers of cardiac hypertrophy and heart failure, remarkably increased in the TAC group compared to the sham group, which was abrogated by PFD and CAP. These data suggested that PFD and CAP attenuated cardiac hypertrophy and dysfunction induced by TAC in vivo.

### 3.2 Pirfenidone alleviated cardiac fibrosis induced by TAC

**FIGURE 2:**
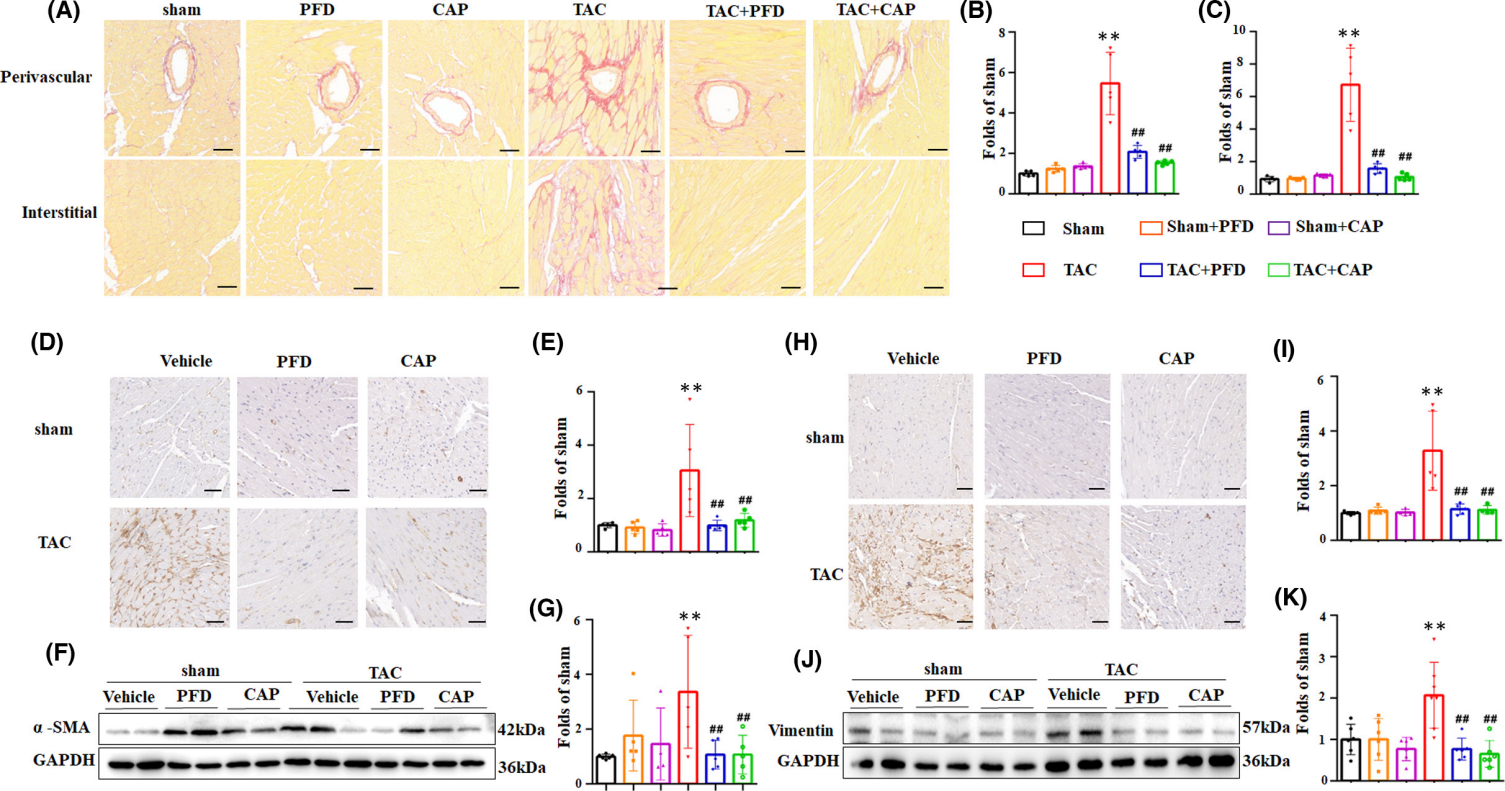
Pirfenidone alleviated cardiac fibrosis induced by TAC in mice. (A–C) Picrosirius red (PSR) staining and quantitative results of cardiac perivascular. (B) and interstitial. (C) fibrosis (*n* = 6 per experiment group). Scale bar = 50 μm. (D, E) Immunohistochemistry (IHC) images and relative expression of α‐SMA in mouse heart sections (*n* = 6 each group). Scale bar = 50 μm. (F, G) Western blotting and relative expression of α‐SMA in mouse heart tissue(*n* = 5–6). (H, I) IHC images and relative expression of Vimentin in mice heart sections (*n* = 6 each group). Scale bar = 50 μm. (J, K) Western blotting and relative expression of Vimentin in mouse heart tissue (*n* = 6). *p < 0.05, ** *p* < 0.01 vs. Sham group; ^#^ *p* < 0.05, ^##^ *p* < 0.01 vs. TAC group

Picrosirius red staining (PSR) (Figure 2A–C) revealed that perivascular and interstitial fibrosis, were dramatically augmented to 5.5 and 6.7 folds respectively, by TAC relative to the sham group. Nevertheless, perivascular fibrosis induced by TAC was mitigated by PFD and CAP to 37.9% and 28.1% of the TAC group, respectively. Meanwhile, interstitial fibrosis was reversed by PFD and CAP to 23.3% and 15.7% of the TAC group. Given that cardiac fibrosis is mainly due to the proliferation and trans‐differentiation of CFs, we measured the content of vimentin and α‐SMA in mouse heart tissue. The result of IHC and statistical analysis (Figure 2D,E) exhibited the expression of α‐SMA was significantly augmented in the TAC group than the sham group, the WB and quantitative cartogram (Figure 2F,G) also manifested the same trend. However, the enhancement of α‐SMA in the TAC mouse heart was reversed by PFD and CAP by approximately 50%. The marker of CFs propagation, vimentin, was increased as evidenced by the IHC and WB results (Figure 2H–K). Similarly, these alterations were alleviated by PFD and CAP after interference for 8 weeks. These data in vivo collectively illustrated that cardiac fibrosis induced by pressure overload was reversed by PFD and CAP through the inhibition of CF differentiation and proliferation.

### 3.3 Pirfenidone inhibited cardiac fibroblasts proliferation and transformation stimulated by AngII in vitro

**FIGURE 3:**
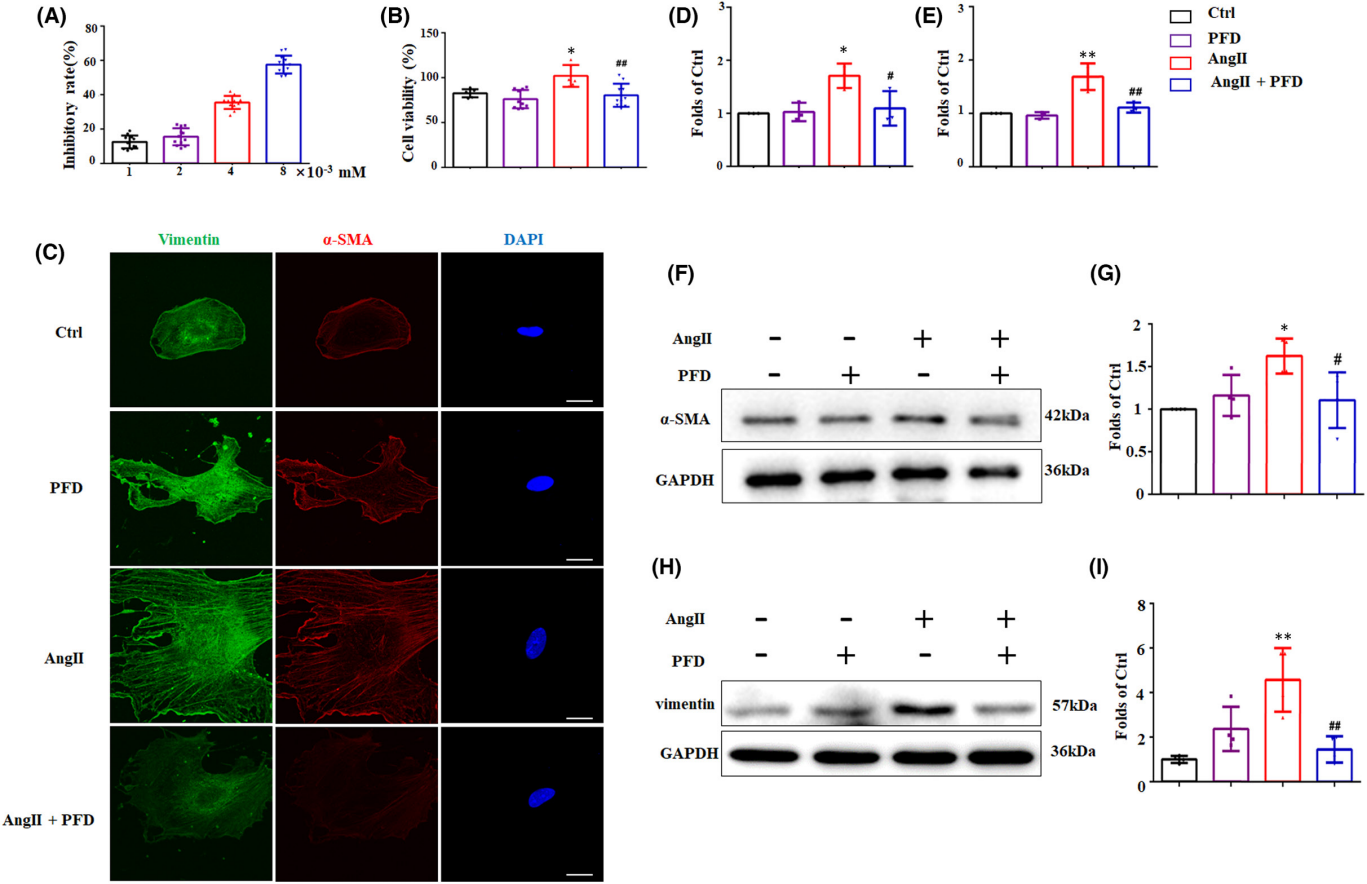
Pirfenidone alleviated cardiac fibroblasts (CFs) proliferation and transformation stimulated by AngII in vitro. (A) Relative inhibitory rate of CFs in different concentrations of PFD (*n* = 6–8). (B) Relative cell viability of CFs at the administration of PFD and AngII (*n* = 6–8). (C–E) Representative immunofluorescent staining photographs and relative fluorescent intensity of Vimentin and α–SMA in the Ctrl, PFD, AngII, AngII + PFD groups in vitro (*n* = 3). Scale bar = 10 μm. (F, G) Western blotting and relative expression of α‐SMA in CFs in vitro(*n* = 4–6). (H, I) Western blotting and relative expression of Vimentin in CFs in vitro (*n* = 4–5). *p < 0.05, **p < 0.01 vs. Ctrl group; ^#^ *p* < 0.05, ^##^ *p* < 0.01 vs. AngII group

In vitro, a pressure‐overloaded model was mimicked by AngII and then administrated with different concentrations of PFD (Figure 3A,B). The viability of CFs was depressed by PFD in a dose‐dependent manner. In addition, PFD inhibited the proliferation of CFs induced by AngII. The expression of vimentin and α‐SMA stimulated by AngII in CFs were evaluated by Immunofluorescent staining (Figure 3C–E). The immunofluorescent intensity of vimentin and α‐SMA in CFs was strikingly increased by AngII stimulation and inhibited by PFD treatment. Western blotting (Figure 3F–I) showed the similar findings, the level of α‐SMA and vimentin in CFs induced by AngII were lessened by PFD treatment. In short, the proliferation and transformation of CFs induced by AngII were largely attenuated by PFD.

### 3.4 Pirfenidone inhibited the TGF‐β1/Smad3 signalling pathway induced by pressure overload

**FIGURE 4:**
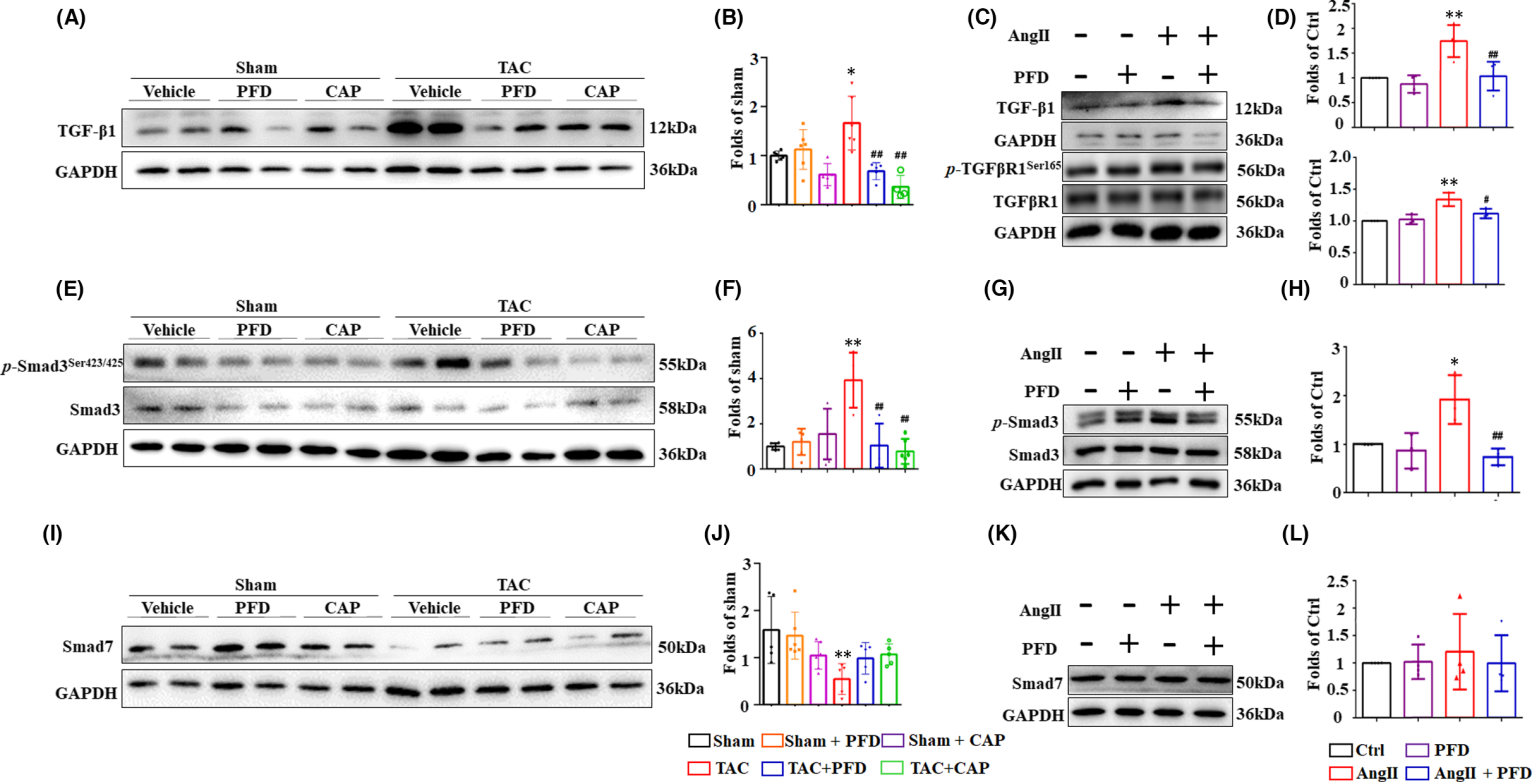
Pirfenidone regulated the TGF‐β1/Smad3 signalling pathway. (A–D) Western blotting and relative expression of TGF‐β1 in mouse heart (*n* = 5–6) and primary cardiac fibroblasts (CFs) (*n* = 4), respectively. (E–H) Western blotting and relative expression of p‐Smad3, Smad3 in mouse heart (*n* = 5–6) and CFs (*n* = 4), respectively. (I–L) Western blotting and relative expression of Smad7 in mouse heart (*n* = 5–6) and CFs (*n* = 4), respectively. *p < 0.05, **p < 0.01 vs. Sham/Ctrl group; ^#^ *p* < 0.05, ^##^ *p* < 0.01 vs. TAC/AngII group

Whether PFD play its anti‐fibrotic role in heart failure via suppressing the TGF‐β1/Smad3 signalling remains unclear. In Figure 4A–D, the expression of TGF‐β1 was significantly increased by TAC and AngII compared to the sham group and the ctrl group in vivo and in vitro, respectively. However, the level of TGF‐β1 was remarkably decreased by PFD. In addition, PFD inhibited the phosphorylation of TGF‐β receptor which was boosted by AngII (Figure 4C,D). Furthermore, given the equal amount of the total Smad3, p‐Smad3 was activated by TAC and AngII in vivo and in vitro (Figure 4E–H). Conversely, p‐Smad3 was decreased by PFD compared to the TAC and the AngII group. These data intensively manifested the activation of TGF‐β1/Smad3 signalling was inhibited by PFD.

A Smad‐dependent signalling pathway is the main mediator of TGF‐β signalling, while smad7 is one of the inhibitory Smads (I‐Smads) that inhibit the expression of Smad3.^19^ We detected the expression of Smad7 induced by pressure overload in vivo and in vitro to show if PFD affects the Smad‐independent pathway. In Figure 4I,J, the expression of Smad7 was remarkably decreased by TAC relative to the sham group in vivo. Whereas PFD did not rescue the expression of Smad7 in the TAC + PFD group relative to the TAC group. Due to the TGF‐β signalling being directly and indirectly regulated by AngII,^20^ CAP diminished the TGF‐β signalling pathway evidenced by the sharp descent of TGF‐β1, smad3 and smad7.

In summary, our study substantiated that cardiac fibrosis induced by pressure overload was attenuated by PFD via inhibiting the TGF‐β1/Smad3 signalling pathway.

## 4 DISCUSSION

**FIGURE 5:**
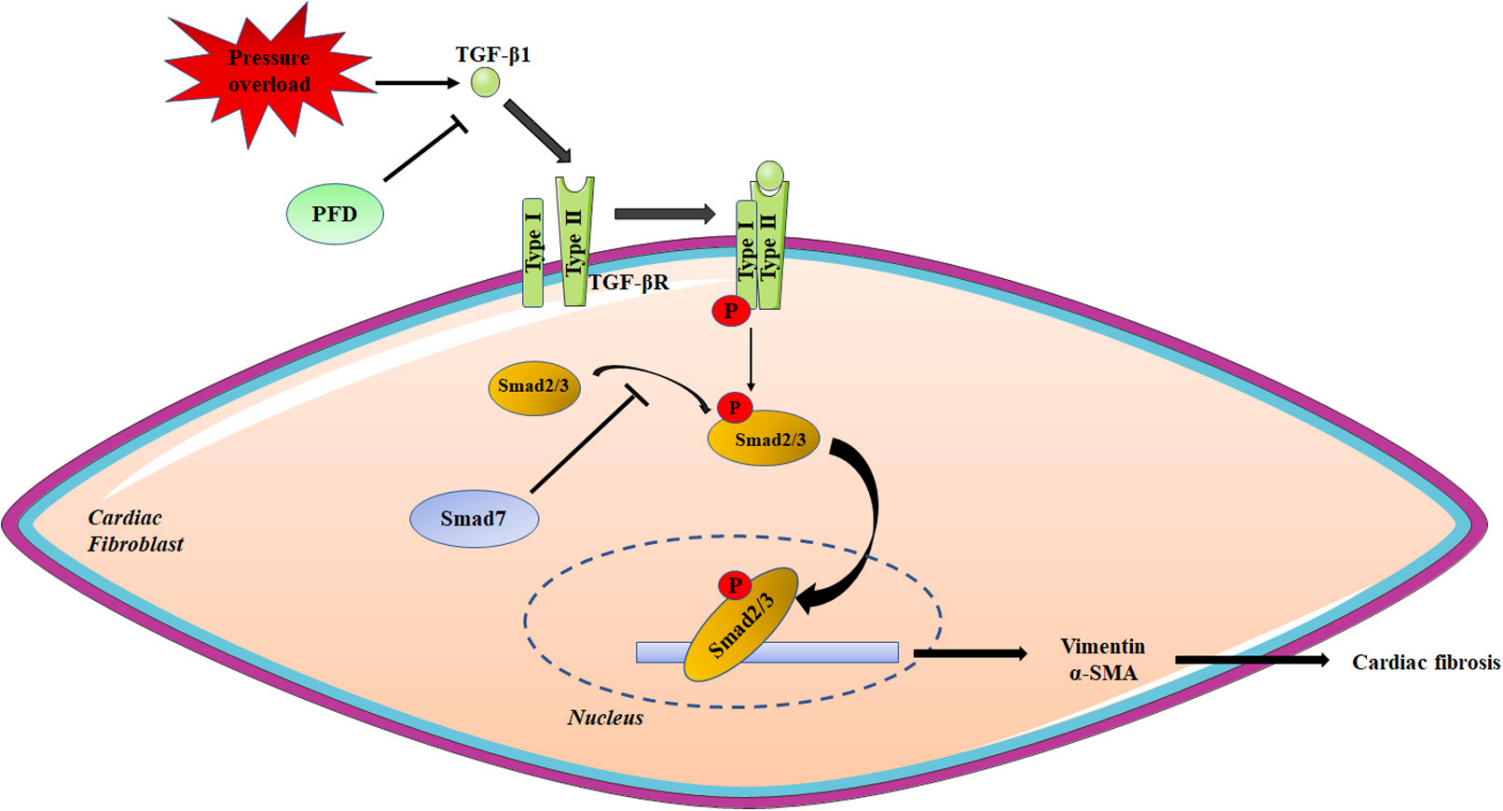
The schematic abstract of PFD attenuating cardiac fibrosis induced by pressure overload via inhibiting TGF‐β1/Smad3 signalling

Our present study showed PFD, a widely used anti‐fibrotic agent for IPF, alleviated cardiac fibrosis induced by pressure overload via inhibiting the TGF‐β1/Smad3 signalling pathway (Figure 5). In this study, cardiac remodelling and heart failure induced by TAC in vivo were alleviated by PFD. Cardiac fibrosis, including perivascular fibrosis and interstitial fibrosis, was diminished by PFD by inhibiting the proliferation and differentiation of CFs. Furthermore, the TGF‐β1/Smad3 signalling which mainly leads to organic fibrosis was suppressed by PFD in cardiac fibrosis induced by pressure overload.

Cardiac fibrosis is predominantly involved in the pathological development of heart failure, leading to deterioration of diastolic function.^21^ Finding effective anti‐fibrosis agents is of great significance to the treatment of heart failure, while with little progress by far. PFD plays a promising role in attenuating organic fibrosis,^8^, ^9^ furthermore, the latest study showed patients with HFpEF benefited from continuous administration of PFD for 52 weeks evidenced by decreased myocardial extracellular volume (MCV).^22^ Our observation from morphological and echocardiography data in vivo validated cardiac dysfunction induced by pressure overload was attenuated by PFD. In addition, cardiac remodelling including cardiac hypertrophy and fibrosis showed in H&E and PSR staining were restrained by PFD, as effective as CAP.

As trans‐differentiation of resident CFs presents the dominant resource of MFs^23^ and impairs the compliance of heart. Apart from the dose‐dependent inhibitory effect of PFD on proliferation of CFs, we examined the expression of α‐SMA and vimentin, the markers of trans‐differentiation and proliferation of CFs. The content of α‐SMA and vimentin stimulated by TAC and AngII were diminished by PFD in vivo and in vitro, respectively.

Wang et al. found PFD inhibited cardiac fibrosis by suppressing the NLRP3 inflammasome formation.^10^ Although a similar finding was shown in our study, we clearly demonstrated an intracellular signalling pathway mechanism, totally different from that they showed. TGF‐β1/Smad3 signalling in activated tissue‐resident CFs is the principal mediator of fibrotic response.^24^ In previous studies, PFD ameliorates pulmonary fibrosis by inhibiting the TGF‐β1/Smad3 pathway.^9^ Besides, cardiac contractility modulation therapy exerted protective effects against myocardial fibrosis via inhibiting the TGF‐β1/Smad3 signalling pathway in rabbits with chronic heart failure (CHF).^25^ These studies showed a pivotal role of the TGF‐β1/Smad3 signalling in myocardial fibrosis. Our presenting data showed PFD played an intensive effect in depressing the activation of the TGF‐β1/Smad3 signalling pathway in pressure‐overloaded mouse hearts and CFs, respectively.

CFs are not only target cells responsible for fibrosis, but also an important source of TGF‐β1. Hence, we examined the releasement of in CFs (Data S1). The result showed that the content of TGF‐β1 was significantly boosted by AngII. However, PFD failed to inhibit TGF‐β1 release. Our data suggested that PFD suppressed intrinsic TGF‐β1/Smad3 signalling pathway of CFs, but cannot attenuate the secretion of TGF‐β1. Considering that the content of intracellular TGF‐β1 was greatly attenuated by TGF‐β1 in CFs, we can still draw the conclusion that PFD could downregulate the level of TGF‐β1. In addition to being involved in the canonical Smad signalling, TGF‐βs are also in Smad‐independent signalling pathways.^12^ Another group of Smad proteins, the I‐SMADs (Smad6 and 7) work in the opposite direction and negatively regulate TGF‐β signalling by competing with the R‐SMADs (Smad1, 2, 3, 5 and 8) for receptor binding.^12^ We evaluated the impact of PFD on the expression of Smad7 in mouse hearts and CFs by pressure‐overloaded stimulation. Smad7 was decreased in TAC mouse heart tissue, leading to attenuated protective effect of Smad7. However, PFD showed no remarkable effect on the expression of Smad7.

In our study, there is no significant distinction between PFD and CAP administration in attenuating cardiac dysfunction and remodelling, whereas CAP sharply inhibited TGF‐β1/Smad3 signalling. This may be due to the AT1R being upstream of TGF‐β1 and regulating canonical and noncanonical TGF‐β1 signalling.^20^ CAP belongs to ACEIs, which downregulates blood pressure to restrain cardiac remodelling and cardiac failure.^26^ CAP was implied to attenuate cardiac apoptosis and hypertrophy induced by TAC, which could be attributed to the inhibition of the Wnt3a/β‐catenin and the JAK2/STAT3 signalling pathway, respectively.^7^ The anti‐hypertrophy role of CAP was validated in our study, however, side effects like coughing, nephrotoxicity and marked hemodynamic impact were inevitable when applying CAP to treating hypertension.^27^, ^28^, ^29^ The present study showed the anti‐fibrotic effect of PFD was not inferior to CAP, which implied great potential in preventing cardiac remodelling and heart failure induced by hypertension without modulating blood pressure. In addition, our study provided a solid foundation for future clinical studies developing novel anti‐fibrotic agents in hypertension.

Although a clear antifibrotic effect and precise mechanism of PFD against cardiac fibrosis induced by pressure overload were unravelled here, there are still some limitations in our study. We validated the beneficial effects of PFD only in the CHF model induced by TAC, however, the mechanism of suppressive effect of PFD to TGF‐β1 was not fully revealed. It was reported that PFD could downregulate the transcription of TGF‐β1 to reduce its protein level. Future studies are called to explore the repercussions of PFD in other pathological models and clinical experiments.

Briefly, our study substantiated that myocardial fibrosis induced by pressure overload, was attenuated by PFD via repressing TGF‐β1/Smad3 signalling. We propose that PFD may become a promising medicament for CHF induced by hypertension. Our work also gives experimental evidences for future clinical trials testing the anti‐fibrosis effect of PFD in heart failure.

## AUTHOR CONTRIBUTIONS

**Na Li:** Conceptualization (lead); data curation (lead); formal analysis (lead); writing – original draft (lead). **Weijian Hang:** Data curation (supporting); investigation (supporting); methodology (supporting); supervision (supporting). **Hongyang Shu:** Investigation (supporting); project administration (supporting); supervision (supporting); validation (supporting); visualization (supporting); writing – review and editing (supporting). **Ning Zhou:** Conceptualization (supporting); funding acquisition (lead); project administration (lead); supervision (lead); writing – review and editing (lead).

## FUNDING INFORMATION

This work was supported by grants from the National Natural Science Foundation of China (grant number 81570261, 82070316) and the Chinese Cardiovascular Association‐Access Fund (2020‐CCA‐ACCESS‐059). The funding sources had no involvement in study design; in the collection, analysis and interpretation of data; in the writing of the report; and in the decision to submit the article for publication.

## CONFLICT OF INTEREST

The authors confirm that there are no conflicts of interest.

## Supporting information


Data S1

## Data Availability

Our data has not been shared previously and is not under consideration for publication elsewhere, in whole or in part.
