# Supplementary material for: Pirfenidone alleviates cardiac fibrosis induced by pressure overload via inhibiting TGF‐β1/Smad3 signalling pathway
Source: J Cell Mol Med. 2022 Jul 21;26(16):4548–55. doi: 10.1111/jcmm.17478 (PMC9357610; doi:10.1111/jcmm.17478)
Supplement: Supplementary file 1 — Data S1 [file JCMM-26-4548-s001.docx]

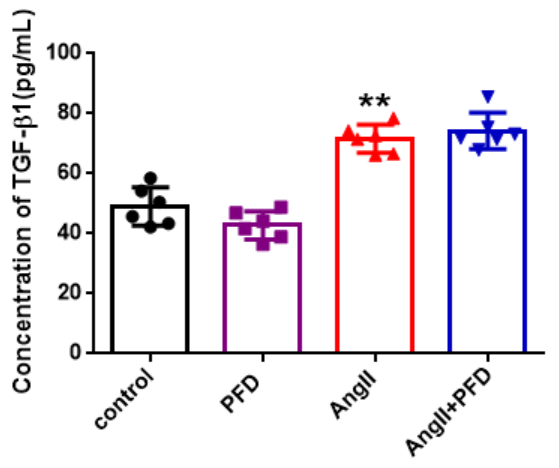


**Concentration of TGF-β1 (pg/mL)**

**0**

**20**

**40**

**60**

**80**

**100**

**AngII +PFD**

**AngII**

**PFD**

**Ctrl**

Supplementary data1 The content of TGF-β1 in the supernatant of CFs stimulated by AngII and PFD.
